# Analysing longitudinal wearable physical activity data using non-stationary time series models

**DOI:** 10.1186/s12966-025-01779-8

**Published:** 2025-07-01

**Authors:** Melina Del Angel, Matthew Nunes, Oliver Peacock, Ewan Cranwell, Dylan Thompson

**Affiliations:** 1 Department for Health, University of Bath, Claverton Down, Bath, BA2 7 AY UK; 2 Department of Mathematical Sciences, University of Bath, Claverton Down, Bath, BA2 7 AY UK; 3 KiActiv®, 1 Duchess Street, London, W1 W 6 AN UK

**Keywords:** Physical activity data analysis, Wearable devices, Trend estimation, Non-stationary time series

## Abstract

**Background:**

Wearable devices have emerged as a new technology for monitoring physical activity over time. Conventional approaches to wearable physical activity data have tended to ignore temporal changes and, instead, have typically analysed summative measures and/or snapshots (e.g., averages over a specific period). In this report, we aimed to develop a novel statistical method to analyse longitudinal physical activity data accounting for the temporal structure in the data.

**Methods:**

This research used secondary data from the Multidimensional Individualised Physical Activity (MIPACT) randomized controlled trial. Physical activity data over the 12-week intervention for 80 participants (28 women) aged between 43 and 70 years old met the criteria for inclusion in this analysis. We modelled the temporal dynamic of each participant using a Trend Locally Stationary Wavelet model, and we introduced the Time in Reference Region of Variability (TIRRV) to assess individual changes relative to baseline.

**Results:**

The analysis of wearable physical activity data poses an important challenge for traditional statistical methods, which often fail to account for dependency between sequential data points and varying characteristics. In this work we demonstrate the effectiveness of a Trend Locally Stationary Wavelet model (TLSW) approach in analysing hourly resolution data from a 12-week intervention, enhancing the understanding of physical activity data, and providing meaningful insights at both individual and group levels. The TLSW considers the time dependency and structure of the data, enabling detailed trend and point-wise confidence intervals analysis. In addition to trends, the newly-developed TIRRV represents a baseline-informed metric to assess the success of individuals and groups over time. The application of these methods produce robust and readily understandable insights about the effect of interventions.

**Conclusions:**

The TLSW-based approach is a novel method for analysing physical activity collected using high-resolution wearable technology. The TLSW trends robustly characterize individual and group behaviour over extended periods of time. This novel approach enables researchers, clinicians, and patients to understand temporal changes in device-measured physical activity data in a way that was not possible previously.

**Supplementary Information:**

The online version contains supplementary material available at 10.1186/s12966-025-01779-8.

## Background

Physical activity plays an important role in the management of multiple health conditions [1, 2]. Patients with pre-existing chronic non-communicable diseases, such as Type 2 diabetes, are recommended to increase their physical activity as a cornerstone of their treatment [3]. Thus, it is critically important to be able to characterise and understand temporal patterns in physical activity, including whether specific individuals or groups of individuals have managed to successfully change physical activity over time.

Wearable devices have emerged as a new technology for monitoring physical activity more objectively and accurately than traditional self-reported methods [4]. Conventional approaches to wearable physical activity data analysis have tended to ignore extended temporal changes and, instead, have typically analysed averages over a specific period, for example, over 7-days before and after a 12-week intervention [5, 6]. However, this overlooks a large amount of potentially important and useful temporal information. Behaviour change is rarely linear and/or constant [7], and a pre-post assessment ignores dynamic changes in behaviour during intervention periods which could provide useful information about intervention outcomes, including a more holistic and complete picture about intervention ‘success’.

Contemporary commercial and research-grade devices can track physical activity for weeks at a very high resolution [8, 9]. Given improvements in device properties (e.g., battery and memory), it is now possible to analyse data over time to understand the dynamic features of physical activity during interventions and in the longer term, rather than only relying on pre-post assessment ‘snapshots’. However, the characteristics of longitudinal data generated from wearable devices comes with a new challenge: how do we analyse large amounts of temporal data to draw meaningful and representative conclusions?

**Fig. 1:**
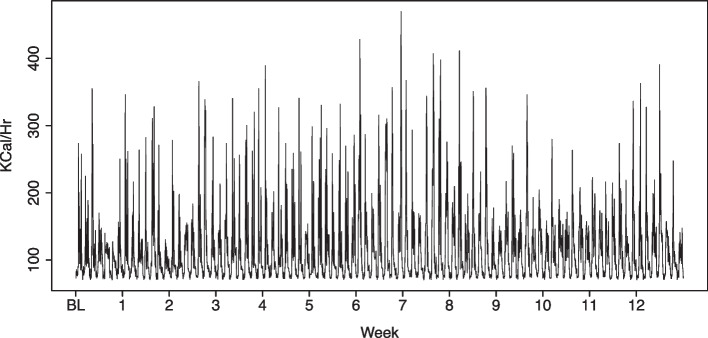
Temporal raw data of participant 80 in the MIPACT study. Hourly resolution data from one participant (patient 80) during the MIPACT intervention [5]. Data is presented at hourly resolution during baseline assessment (BL) and over the following 12-weeks

For a given individual, if data from wearable devices are captured at minute-by-minute resolution, then this represents > 120,000 data points over a typical 12-week intervention. As can be seen in Fig. 1, it is very challenging to determine temporal changes or trends in physical activity through visual exploration of the data, even when visualised at hourly resolution. The sequential way that wearable physical activity data is collected produces temporal series with a longitudinal correlation structure among adjacent points. This inherent temporal dependency limits the use of traditional statistical methods that typically assume independent observations. Moreover, this correlation structure tends to change over time, and consequently the statistical properties in the observed data can be dramatically different at different intervals of the monitoring period. Thus, simply analysing raw physical activity data numerically for either an individual or a group of individuals over time ignores the time-dependent dynamics and would be unlikely to provide a meaningful picture of physical activity measures over time.

The development of new tools to analyse longitudinal PA data could be useful in medical research for the assessment of interventions, and to examine the interaction between longer term trends in physical activity and the pathogenesis or progression of chronic diseases. Such tools could also be useful in clinical practice, allowing clinicians to monitor patients’ activity levels over time, and to give them tailored advice as part of primary and/or secondary prevention strategies. Furthermore, if the output from these new tools can be made accessible and integrated into user-friendly applications, patients themselves could benefit through an improved understanding of their physical activity over time, promoting adherence to interventions and programmes.

In addition to the non-linear nature of physical activity behaviour change over time, physical activity data tend to have cyclical patterns produced by individuals’ habits and routines, such as the case of day-night shift that typically results in higher levels of physical activity during daytime, and lower levels during the night, or the activity levels during weekdays versus weekends that tend to be different due to work and leisure time. Another important feature to consider is that wearable devices can produce noisy data [10], and thus robust models are needed to extract meaningful insights and mitigate the effect of noise.

Our aim is to develop novel statistical methods to analyse longitudinal physical activity data. Our approach uses Time Series Analysis, a set of statistical models designed to account for the temporal dependency in continuous ordered data [11, 12]. One of the advantages of using time series in the analysis of wearable data is that the underlying components of individuals’ physical activity such as trend, error and cyclical components can be identified and analysed separately in full resolution. Potentially, time series analysis could be used at an individual level, supporting more realistic decision-making by health professionals and patients, as well as by researchers hoping to characterise the true effect of their interventions.

In the context of physical activity, the sequential records of energy expenditure constitute a series for each individual. When the characteristics of the series, such as trend or variability, vary with time, they are described as non-stationary time series [11]. Because the physical activity data trend is assumed to change over the intervention period, and because variability changes with time, a non-stationary time series model is more suitable for wearable data. Locally Stationary Wavelet Process (LSW) can model time series where variance structure changes with time but, equally important in this case, LSW effectively filters out meaningless information from the data to capture the underlying components of physical activity, even in the presence of non-stationarity [13].

Locally stationary time series models have been effectively used in analysis of non-stationary time series such as sleep patterns in young infants [14], circadian rhythms in plants [15], and brain activity [16]. However, as far as we are aware, there has been no prior attempt to apply time series models which account for both trend and variance changes to physical activity data. Thus, in this analysis, we will examine whether methods-based on the Trend Locally Stationary Wavelet (TLSW) model can be used to characterise individual and group physical activity data over time. As proof of concept, we focus on sedentary time as a key and complex physical activity-related outcome.

## Methods

**Fig. 2:**
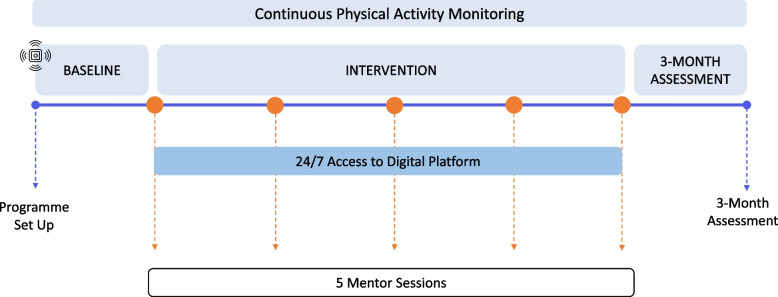
A schematic of the Intervention arm of the MIPACT study. Physical activity data was collected continuously for 14 weeks [5]. Patients were blinded to their data during the baseline period after which they had access 24/7 to a web App to self-monitor their physical activity and sedentary time data

This analysis uses data from the intervention arm of the Multidimensional Individualised Physical Activity (MIPACT) randomized controlled trial [5, 6]. The MIPACT study recruited patients at medium risk of cardiovascular disease and/or type II diabetes via primary care in the United Kingdom. As shown in Fig. 2, patients in the Intervention arm continuously wore a physical activity device for 14 weeks (BodyMedia Core, BodyMedia Inc., Pittsburgh, PA). This multi-sensor device is worn on the upper arm and has been extensively validated [17–19]. Participants were asked to wear the device 24 h per day except when showering or in contact with water, but they were also allowed to remove it at night for comfort if required [6]. The sensors in the device establish when it is on and off body to derive wear time. During the first seven days (baseline), patients were blinded to their data but, subsequently, they were provided access to a digital platform to self-monitor their physical activity [5, 6] for 12 weeks. In this study, we analyse data from the first 13 weeks, excluding week 14 as the intervention concludes at the end of week 13.

The MIPACT study provides a useful dataset to explore novel methods for the analysis of longitudinal wearable data. For this analysis, from the 121 participants allocated to the intervention arm who completed the 3-month assessment [6], we included individuals with at least 10 complete weeks of data during the 12-week intervention period, where a week was considered complete if it had at least 6 complete days; and a day was considered complete when at least 80% of the waking hours were recorded by the device (768 min excluding 8 h of sleep time). This resulted in a final sample size of 80 for the current analysis. For these participants, the average wear time during typical waking hours (6:00am to 10:00 pm) was 89%±7% (mean±SD) over the 13-week period. Thus, the longitudinal data used in the present analysis represents a nearly complete dataset collected over a prolonged period of time (approximately 3 months).

### Time-series for physical activity data analysis

**Fig. 3:**
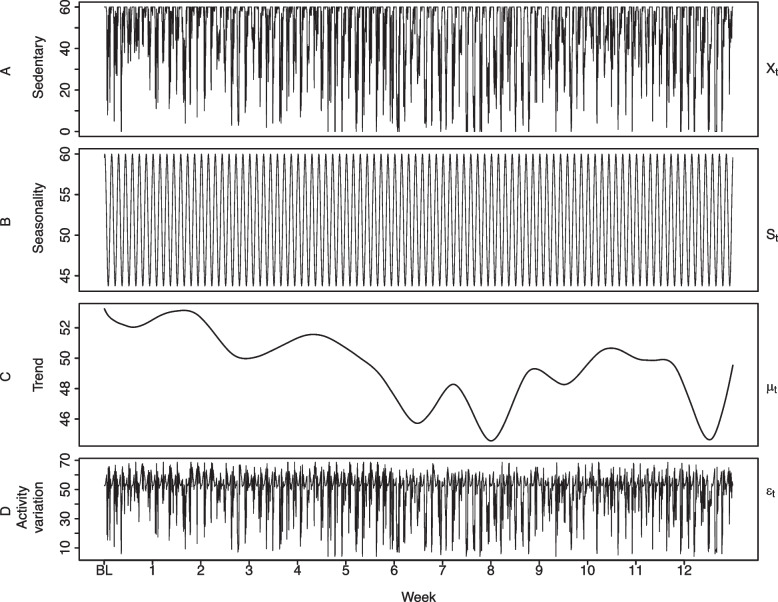
Sedentary mathematical components for one example individual (participant 80). The y-scale in the four panels is minutes per hour. **A** Observed sedentary time per hour. **B** cyclical component derived from the day-night shift and estimated with Fourier series. **C** estimated trend using TLSW. **D** activity variation captured using TLSW

We calculate the number of sedentary minutes per hour with sedentary time defined as time < 1.8 METs using this device [6]. One MET represents basal metabolic rate. Missing data were assigned estimated basal metabolic rate [20]. Over the 13-week period, this aggregation results in a sequence of 2,184 hourly-resolution ordered values per participant. These ordered values constitute an individual’s time series for sedentary time and is denoted as ${X}_{t}$. The produced time series for one MIPACT individual is illustrated in Fig. 3A*.* To explore the temporal dynamic of the series, a TLSW model is fitted for every participant. Analysing these data using conventional approaches (e.g. one-way repeated measures ANOVA), does not provide a clear perspective regarding changes in sedentary time (see Supplement online).

### Components of physical activity

Physical activity data are affected by various sources of variation, and this variation can be identified using a time series decomposition. A time series decomposition is a standard statistical method from which the time series is broken into the main underlying components: seasonality, trend, and variability.

Seasonality, denoted as ${S}_{t}$, refers to the cyclical patterns in the data, and in the context of physical activity, is produced from habits and regular repeated activities. The most predominant seasonality in the data is the day-night shift which is manifested as a decrease in sedentary time during wake time and an increase in sedentary time during sleep. This pattern is repeated every day, creating a 24-h cycle in the data. Additionally, the series can be influenced by one or more other seasonal factors. For example, the transition between weekdays and weekends could produce another cycle every week. Moreover, seasons of the year could induce another cycle for longer monitoring periods, as individuals may change their habits between seasons (spring, summer, winter, and autumn). These cycles are assumed to be independent among individuals, as individuals do not necessarily share routines. The cyclical components are estimated for individual time series (number of minutes spent by the individual in sedentary time throughout the monitoring window) using a Fourier series [21]. The estimated seasonality for one MIPACT participant is illustrated in Fig. 3B, notice how the 24-cycle dominates the seasonality that goes up as sedentary time increases during nights and down during daytime.

The second component in the data is the trend, denoted as ${\mu }_{t}$. The trend is the change in sedentary time captured by the time series model when other influencing factors are removed. In this context, the trend can be seen as temporal representation of the changes and fluctuations in sedentary time. In Fig. 3C, the estimated trend using a TLSW model for one participant is shown. The trend characterises the longitudinal changes in sedentary time and also provides an estimation of the time spent by the individual in sedentary time at every timepoint.

Finally, Fig. 3D illustrates the ‘activity variation’, denoted as ${\varepsilon }_{t}$ and estimated using the TLSW model [13]. The activity variation, also called random error or noise [22], captures the variability that cannot be explained by the seasonality or trend. It captures irregular activities and unpredictable variation in the data.

### Individual point-wise confidence intervals

Curve trends characterize the temporal dynamics of the data series. They can identify the direction and magnitude of changes in the presence of high variability. This aspect is especially relevant when dealing with high frequency data, as is usually the case with wearable devices.

**Fig. 4:**
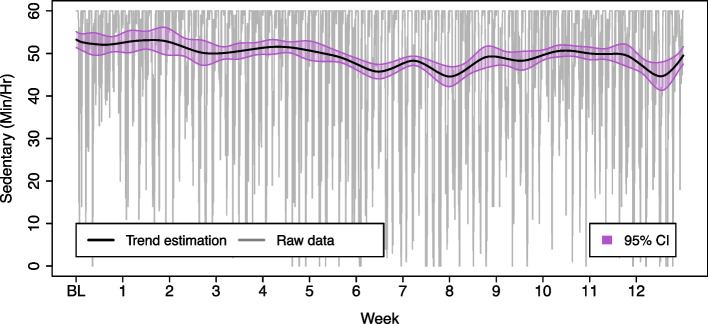
Trend estimation using TLSW for Sedentary time. The trend curve $({\mu }_{t})$ is overlapped to the observed data $({X}_{t})$. Pointwise 95% confidence intervals are represented in the shaded region

In Fig. 3C, an example of the estimated ${\mu }_{t}$ of sedentary time for one participant is shown. The decreasing trend during weeks 1 to 6 suggests a reduction of sedentary time and reflects a possible positive impact of the intervention towards the end of the programme. This change could not be appreciated from ${X}_{t}.$ In Fig. 4, the ${\mu }_{t}$ is presented as a solid line overlapped to ${X}_{t}.$ One can observe that, because of the amount of noise in the ${X}_{t},$ it would be difficult to visually determine if the individual is increasing or decreasing their sedentary behaviour. With these types of data, line plots become unreliable because the visual effect hides the true tendency and leads to misinterpretations. Descriptive statistics would also fail to provide a fair assessment, because the noise will affect central tendency measures. In contrast, the trend curve can be easily interpreted, and analyses based on the trend remain not easily affected by noise and outliers.

Another advantage of using time series for analysing wearable data is that confidence bands can be estimated for the data trend (Fig. 4). McGonigle et al*.* [13] describes a way to obtain simultaneous confidence intervals as a function of the spectral estimate by making use of the multivariate normal distribution.

### Time in reference region of variability (TIRRV)

**Fig. 5:**
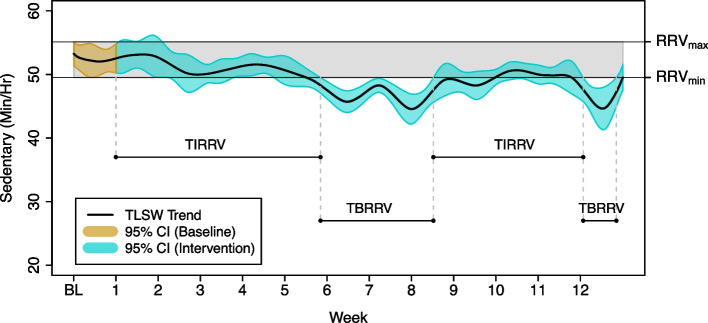
Time in RRV for one individual (participant 80). The estimated PA trend is presented along with its 95% confidence band. The RRV delimited by ${\text{RRV}}_{\text{min}}$ and ${\text{RRV}}_{\text{max}}$ is shadowed in grey. Two intervals of significant decrease in Sedentary time are identified by the method, starting in weeks 6 and 12

We define the Time in Reference Region of Variability (TIRRV) as the proportion of time over the 12-week intervention that an individual spends close to their observed baseline assessment. To assess if an individual behaves similarly to their baseline, we introduce the concept of Reference Region of Variability (RRV) as the variation range in the trend during the baseline week, when the individual was free-living but blinded to their physical activity and sedentary time data (Fig. 5). The RRV is determined by the interval between the minimum and maximum points of the confidence bands of the trend estimation during the first week. We define ${\text{LL}}_{\text{t}}$ and ${\text{UL}}_{\text{t}}$ as the lower and upper limits of the confidence band of a given individual at time $t = \{1, 2, \cdots ,n\}$ respectively. The RRV is given by $\left[{\text{RRV}}_{\text{min}},{\text{RRV}}_{\text{max}}\right]$ where:

$${\text{RRV}}_{\text{min}}= \underset{\text{t}=\{\text{1,2},\cdots ,168\}}{\text{min}}{\text{LL}}_{\text{t}}$$

$${\text{RRV}}_{\text{max}}= \underset{\text{t}=\{\text{1,2},\cdots ,168\}}{\text{max}}{\text{UL}}_{\text{t}}$$

Using a range (RRV) to represent the baseline is more sensible than using a single threshold value because it is more realistic to assume that activities are not completely fixed and stable. Once the RRV has been defined for each individual, the TIRRV can be obtained as the proportion of time that the confidence bands overlap the RRV over the intervention period, and can be computed as follows:

$$TIRRV= \left(\frac{1}{2016}\right)\left(\sum\limits_{t=\{\text{169,170},\cdots ,2184\}}1\left\{{{RRV}_{min}\le LL}_{t}\le {RRV}_{max}, U{L}_{t}\ge {RRV}_{max}\right\}+1\left\{{{RRV}_{min}\le UL}_{t}\le {RRV}_{max}, L{L}_{t}\le {RRV}_{min}\right\}+1\left\{{{RRV}_{min}\le LL}_{t}, {UL}_{t}\le {RRV}_{max}\right\}+1\left\{{{RRV}_{min}\ge LL}_{t}, {UL}_{t}\ge {RRV}_{max}\right\}\right)$$

where $1\left(\cdot\right)$ is the indicator function, and 2016 is the number of hours in the intervention period. Notice that the length of the assessment period (12 weeks = 2016 h) varies according to the study setting, but this could be adjusted to shorter or longer intervals for assessing other time frames.

In addition to the TIRRV, two more metrics can be derived by using the RRV: (i) the Time Above Reference Region of Variability (TARRV) and (ii) Time Below Reference Region of Variability (TBRRV). The proportion of Time Below Reference Region of Variability (TBRRV) represents the proportion of time over the 12-week intervention an individual decreased their sedentary time in contrast to their baseline, and is obtained by summing the number of times the confidence band was strictly lower than ${RRV}_{min}$:

$$TBRRV= \left(\frac{1}{2016}\right)\left(\sum_{t=\{\text{169,170},\cdots ,2184\}}1\left\{U{L}_{t}<{RRV}_{min}\right\}\right).$$

Finally, the proportion of Time Above the Reference Region of Variability (TARRV) represents the proportion of time over the 12-weeks that an individual spent with increased sedentary time, and is obtained by summing the number of times that the confidence bands were strictly above the ${RRV}_{max}$ divided by the number of time points in the intervention interval:

$$TARRV= \left(\frac{1}{2016}\right)\left(\sum_{t=\{\text{169,170},\cdots ,2184\}}1\left\{L{L}_{t}>{RRV}_{max}\right\}\right).$$

The joint analysis of these three metrics provides a comprehensive understanding of the individual’s progress during the intervention. The use of TLSW allows the estimation of point-wise confidence intervals with a pre-defined level of confidence $\left(1-\alpha \right)100\%$. Hence, RRV can be used to assess whether changes are significant [23]. If TIRRV is greater than zero, it is indicative that sedentary time is significantly different than the baseline at a predefined significance level. If the change is positive or negative, it will be reflected in changes to TARRV and TBRRV, respectively. If TARRV > TBRRV, this indicates that the individual has increased Sedentary time. If TBRRV > TARRV, then this indicates that the individual has decreased Sedentary time.

In the example in Fig. 5, TIRRV = 71.1%, TBRRV = 28.9%, and TARRV = 0%. This means that, for 71.1% of the intervention period, the participant performed similarly to baseline, whereas 28.9% of the time they reduced time spent sedentary.

The TIRRV can also be calculated in different interval lengths, for example, at six-week intervals for comparing the first against the second half of the programme, or weekly intervals for a more detailed view.

### Group confidence intervals

When analysing group data, a trend estimation can be obtained for each individual, as well as the group mean trend along with its confidence bands [21]. It is important to note that the variance estimation of each individual may vary at every point in time and does not necessarily remain equal. Considering unequal variances among individuals throughout the interval, the pointwise confidence intervals of the mean trend can be obtained as follows:

$$\widehat{M}\left(t\right)\mp {q}_{1-\frac{\alpha }{2}}\sqrt{ \frac{\sum_{i=1}^{N}{\hat{\sigma} }_{i}^{2}(t)}{N}},$$

where ${\hat{\sigma} }_{i}^{2}(t)$ is the estimated variance using TLSW of individual $i$ at point $t$, and $\widehat{M}\left(t\right)$ is mean trend group.

## Results

We applied TLSW-based methods for evaluating temporal changes in the time spent in sedentary behaviour. The results are presented from individual and group perspectives. At the individual level, each method was applied to every participant (*N* = 80). To highlight the resulting metrics, we present findings from three participants illustrating diverse performance outcomes (participant codes 20, 155, and 163), as well as whole group level data. The proportion of participants who showed positive, negative and no change in their trend are also presented as a proposed way to assess intervention effectiveness. The results are presented in this way to demonstrate the adaptability of the methods to summarize performance, offering both a comprehensive overview of intervention effectiveness, and detailed insights into individuals’ behaviours.

**Fig. 6:**
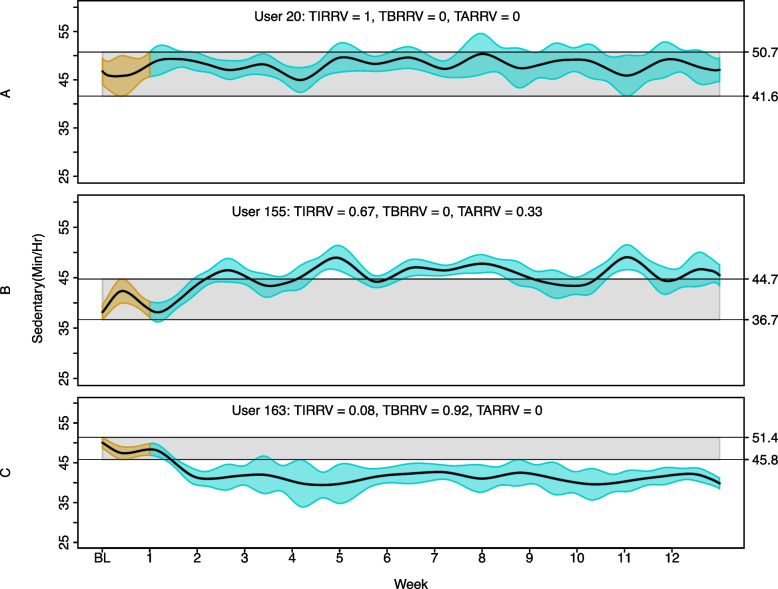
Time series approach to sedentary time data. **A **Participant 20 did not show an important change during the intervention. **B** Participant 155 showed an increase in the time spent in sedentary behaviour around week 5, 7, 8 and 11. **C** Participant 163 reduced their time spent in sedentary behaviour for 92% of the time during the 12-week intervention

The three metrics obtained with the time series approach (TIRRV, TARRV, and TBRRV) for the three cases are presented at the top of Fig. 6(A-C). Figure 6A shows the sedentary trend for one participant who did not show any change during the intervention period (TIRRV = 1). Figure 6B and C show examples for individuals who present negative and positive changes, respectively. The participant shown in Fig. 6B spent 67% of the intervention period similar to baseline (TIRRV = 0.67), and 33% of the time with increased sedentary behaviour (TARRV = 0.33). The example participant shown in Fig. 6C spent 92% of the intervention with significantly lower sedentary time compared to their baseline (TBRRV = 0.92).

**Fig. 7:**
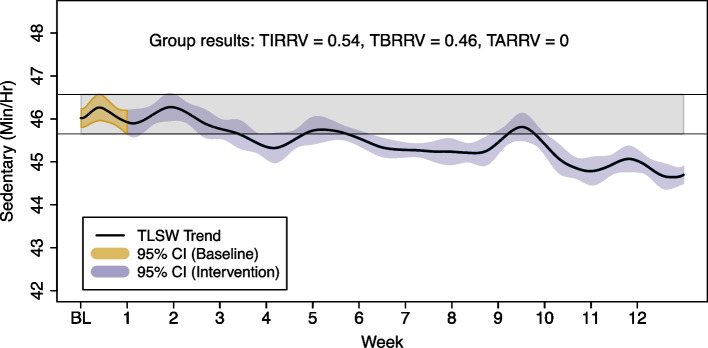
Group time series approach to sedentary time data. Group behaviour: mean trend obtained from the 80 individual trends (obtained with TLSW). Confidence intervals of 95% of confidence were obtained assuming unequal variances

The group TLSW trend and TIRRV is illustrated in Fig. 7. These results indicate that the group spent 46% of their time with reduced time in sedentary behaviour over the 12-week intervention period (TBRRV = 0.46). The reduction in sedentary time was generally from around week 6 until the end of the programme, with a slight deviation from this trend between week 9 and 10. There was a progressive separation of the TLSW trend from the lower limit of the RRV ${\text{(RRV}}_\text{Min}=45.7)$, suggesting a continued reduction in sedentary time throughout the programme. At the group level, there was no indication for an increase in sedentary time at any point in time (TARRV = 0).

**Fig. 8:**
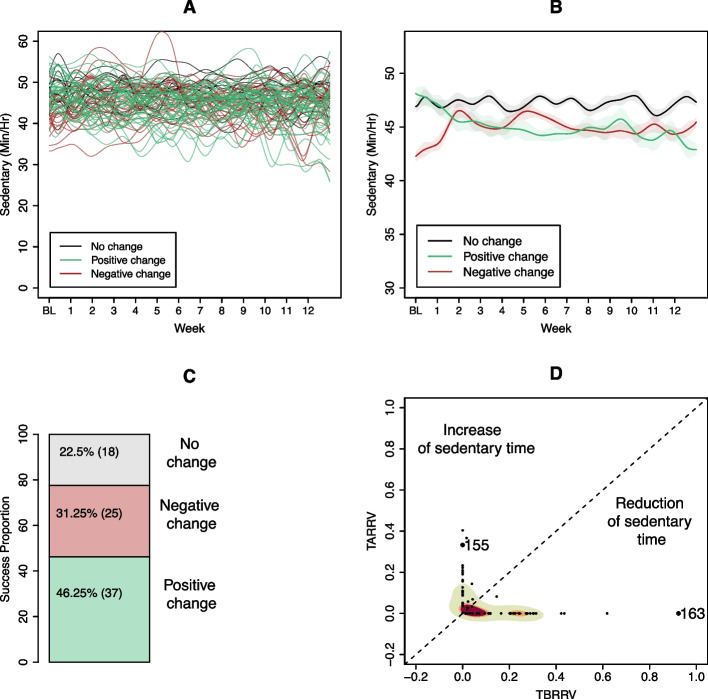
Temporal results of time series approach. **A** Individual trend estimations (*N *= 80): green denotes individuals that had a positive outcome (TBRRV > TARRV), red denotes individuals with negative outcome (TARRV > TBRRV), and black denotes individuals with no change (TIRRV = 1). **B** Mean curve trend for each sub-group: positive outcome (*N* = 37), negative outcome (*N* = 25), and no change (*N* = 18). **C** Barplot representing the proportion of individuals into each category according to their outcome. **D** Bidimensional density plot of individuals who presented a change (*N* = 62). Region with higher density is shaded in red, transitioning through orange and green in areas of lower density. Most participants who changed their sedentary time reduced it (TBRRV > TARRV). Black points with number legends correspond to the users in Fig. 6 that showed a change compared to their baseline

TLSW trends of all participants are presented in Fig. 8A, with the trends colour-coded as green, red, and black to signify decreases, increases, and no change in sedentary behaviour during the intervention, respectively. Identifying a group pattern from this plot is challenging due to the overlapping trends. Thus, in Fig. 8B and 8 C, the average trend for sub-groups (B) and their relative proportions (C) are shown. Almost half the participants (*N* = 37) reduced their sedentary time, with TBRRV > TARRV (Fig. 8C). To understand the magnitude of changes, Fig. 8D provides a bidimensional density plot for the proportion between TARRV and TBRRV for each individual. The farther an individual falls from the origin (coordinates TBRRV = 0, and TARRV = 0), the greater the positive or negative change.

## Discussion

Wearable physical activity data is temporal in nature due the sequential way it is collected. Every datapoint is recorded in a specific time and is not independent from other records but, rather, a correlation between adjacent points exists. In addition, statistical attributes in the data such as variance and mean typically change over time (non-stationarity) and exhibit high noise. These characteristics represent major challenges in the statistical analysis of wearable physical activity data as traditional aggregation methods assume the observed datapoints to be independent, ignoring correlation structures, and failing to identify temporal patterns in the data (e.g. cycles/seasonality). Thus, new approaches are required to analyse longitudinal physical activity data.

In the current report, we show that a TLSW approach can be used for estimating trend performance in a group of patients undergoing a 12-week intervention. In contrast to traditional approaches, the time-series approach considers the time correlations and time-varying attributes (non-stationarities) in the mathematical models to provide meaningful insights at both an individual and group level. These novel statistical methods and approaches for the analysis of longitudinal device-measured physical activity data open up exciting opportunities for researchers, healthcare professionals, and the general public.

As far as we are aware, this is the first attempt to use a TLSW approach for device-measured continuous data. TLSW approaches have been used for other complex temporal high frequency data such as circadian rhythms [15] and sleep trends [24]. The present proof-of-principle analysis demonstrates that a TLSW approach can be usefully developed and implemented for longitudinal physical activity-related data (sedentary time). One specific advantage of TLSW-based approaches is the removal of seasonality which, in the context of sedentary behaviour, removes the sometimes-problematic issue of sleep which is technically sedentary based on energy expenditure but not considered negative from a health perspective. With the advent of devices with improved battery life and data management/storage, the ability to model and understand physical activity over time will become increasingly important. Collectively, we hope that these approaches will enable researchers and/or clinicians to understand temporal changes in device-measured physical activity data in a way that was not possible previously.

We also demonstrate that this method can be used to characterise data from specific individuals as well as groups. In contrast to other approaches, we show that trends along with point-wise confidence intervals provide detailed information of an individual’s sedentary behaviour at hourly resolution. This information could be helpful for clinicians or healthcare professionals who are working with specific individuals or patients, helping them to provide better support and guidance and leading to more effective therapies. Furthermore, this information could be helpful for the patients themselves, enabling them to track and monitor trends in their data, potentially even in real-time.

In addition to trends, summary metrics can be obtained by using this approach to robustly characterise individual and group performance over time. The TIRRV, TARRV, and TBRRV were developed as baseline-informed metrics to assess the success of individuals over the course of the 12-week programme. We show that individual participant TLSW trends, along with their RRVs, are a useful way to compare performance among individuals. For instance, participant 20 exhibited minimal change throughout the intervention, while participants 155 and 163 showed different and opposing changes. At the group level, the TIRRV, TARRV and TBRRV are a methodology that accounts for individual and group variation and have the characteristic of being easy to interpret, as they are proportions that also complement between them (TIRRV + TARRV + TBRRV = 1). Using these approaches, it would be possible to obtain other characteristics of the time series data, for example, whether specific periods (e.g., around key features such as mentor meetings or other interventional components) are associated with changes at an individual or group level. Of course, these estimated changes relative to baseline are only as robust as the baseline assessment – and further work is required to understand what measurement period represents an adequate assessment of baseline.

In the current proof-of-principle analysis, we have focussed on sedentary time per hour as a key outcome that is challenging to analyse using existing statistical approaches. Sedentary time is based on thresholds of energy expenditure that are individualised because they are expressed relative to estimated resting metabolic rate. The current analysis is based on estimated sedentary time from a robust multi-sensor device worn on the upper arm that has been widely validated [17–19]. Further work is needed to examine the use of these methods with different devices worn in different locations (e.g., wrist-mounted accelerometers), and analysed at different resolutions. In addition, it is likely that this novel approach can also be used to analyse other physical activity dimensions such as moderate-to-vigorous intensity physical activity, or to analyse minute-by-minute energy expenditure or other raw accelerometery data. It may be valuable to use time-series approaches to analyse multiple metrics simultaneously to assess their coevolution over time and gain insights into interactions (e.g. sedentary time, MVPA, or others). It is important to consider that other dimensions of physical activity may have different levels of error and completeness to sedentary time as used in the present analysis, and which may therefore require modifications to TLSW modelling approaches. Hence, further research is required to confirm that these approaches work with longitudinal physical activity related data with different inherent structures.

The current study used MIPACT data from the intervention arm to develop and implement new mathematical models. The strengths of this dataset include the use of a widely validated and robust multi-sensor measurement device [17–19], very high wear time over an extended period (~ 90% over 13 weeks), and a sample of diverse patients recruited via primary care. The absence of data for the control group during the 12-week intervention period in the MIPACT study means that it is not possible to draw strong conclusions about changes during the MIPACT intervention based on the current analysis alone. However, the combination of improved device technical capability and affordability, coupled with the proposed improved statistical methods for handling longitudinal data, mean that it should be possible in the future to model data for both control groups (blinded) and intervention groups to robustly characterise dynamic and temporal responses to interventions in randomised controlled trials.

The technical knowledge required for this analysis is a potential limitation, as it requires statistical, mathematical, and programming knowledge to be performed. However, user-friendly interfaces could be developed to overcome this limitation and make the approach more accessible to researchers, healthcare professionals and potential patients or the public. 

## Conclusions

We demonstrate that a TLSW-based approach is a novel method for analysing physical activity data measured using high resolution wearable technology. The TLSW trends are a statistically robust and intuitively easy method to characterize individual and group behaviour over extended periods of time, moving beyond traditional pre-post mean analyses.

## Supplementary Information

Supplementary Material 1.

## Data Availability

All the individual participant data is available, after deidentification, through the University of Bath repository, 10.15125/BATH-01416.

## Abbreviations

MIPACT: Multidimensional Individualised Physical Activity
LSW: Locally Stationary Wavelet process
TLSW: Trend Locally Stationary Wavelet process
RRV: Reference Region of Variability
TIRRV: Time in Reference Region of Variability
TBRRV: Time Below Reference Region of Variability
TARRV: Time Above Reference Region of Variability
