# Supplementary material for: Analysing longitudinal wearable physical activity data using non-stationary time series models
Source: Int J Behav Nutr Phys Act. 2025 Jul 1;22:88. doi: 10.1186/s12966-025-01779-8 (PMC12220009; doi:10.1186/s12966-025-01779-8)
Supplement: Supplementary file 1 — Supplementary Material 1. [file 12966_2025_1779_MOESM1_ESM.docx]

**Aggregation methods for wearable physical activity data analysis**

**A Week-averaged visualizations**

Two examples of visualizations of the weekly average of sedentary time are presented in Figure 1. Notice that in the right panel, the dotted line represents the group average of sedentary time across all weeks, and it falls within all confidence intervals. This is an indication that there may not be a statistically significant difference in sedentary time across weeks.

**
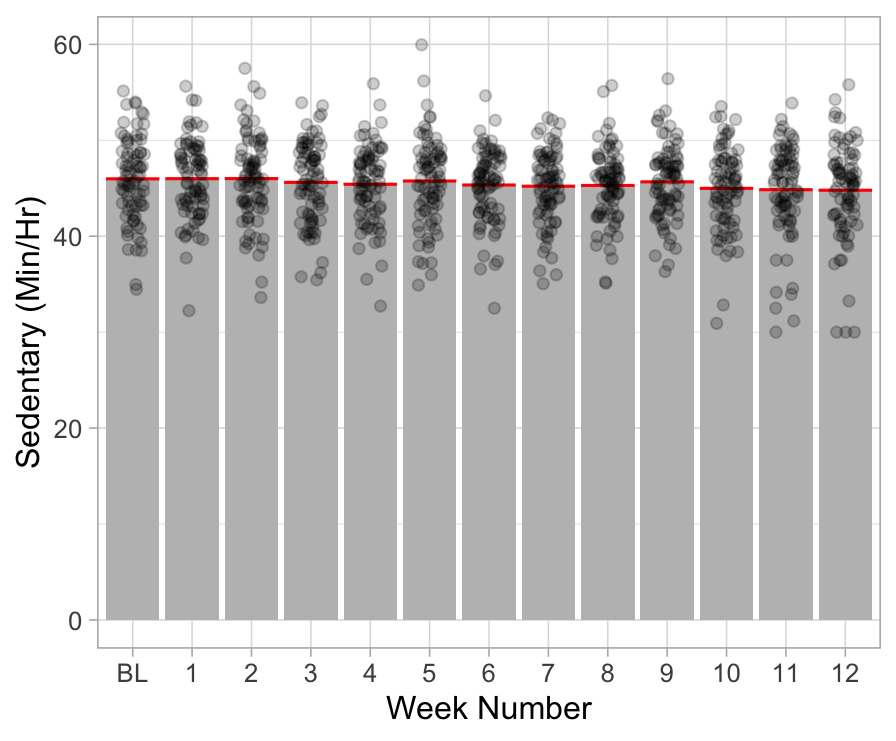
** **
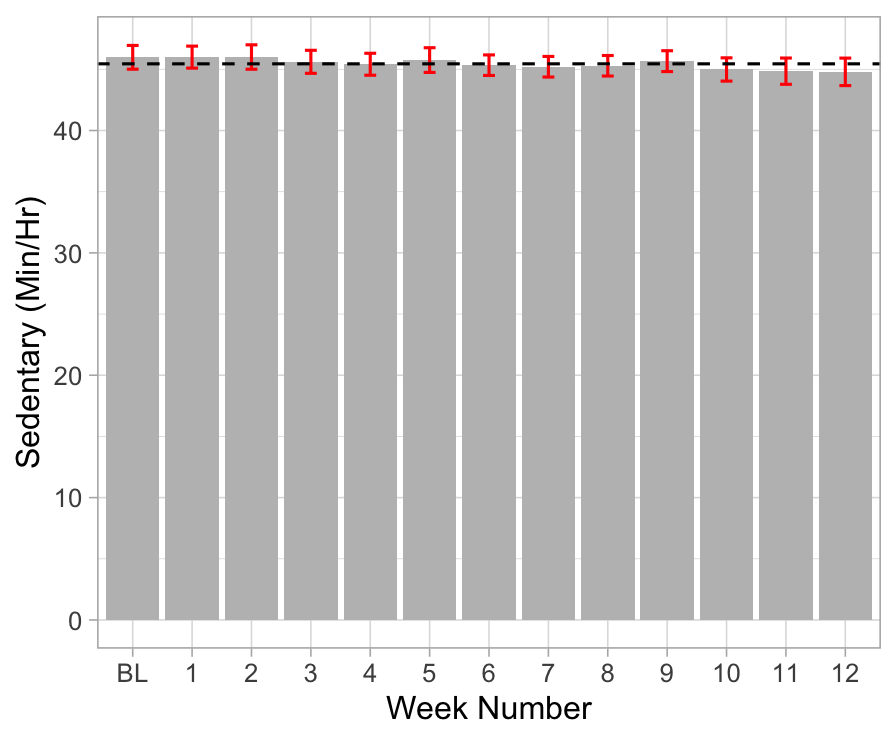
**

Figure 1. Left panel: Black points represent the weekly average of hour-resolution sedentary time by individual (N=80), barplot represent the group average per week. Right panel: Barplots represent the group average of sedentary time per hour (N=80). Red confidence intervals for the mean of 95% of confidence are represented in red. Black dotted line represent the average of sedentary time throughout the 13 weeks (Avg = 45.45).

**B Repeated measures ANOVA**


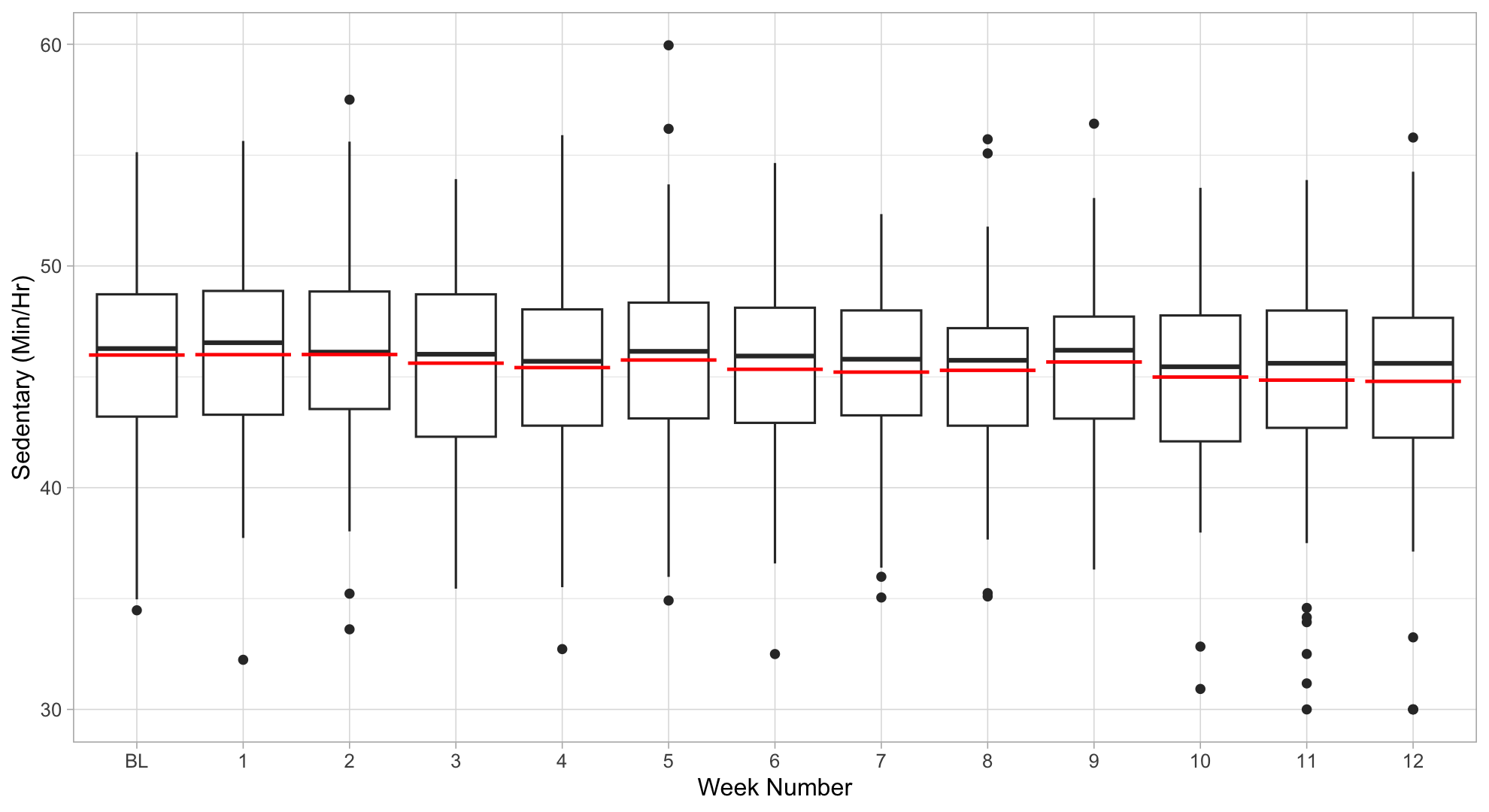


Figure 2. Boxplots of the sedentary time week average for each patient. Horizontal black lines in the boxes represent the group mean. Horizontal red lines in the boxes represent group median.

The one way ANOVA using Greenhouse-Geisser correction indicates that there was an effect of time (p=0.014) but post-hoc pairwise t-tests with Bonferroni correction was unable to identify any differences between weeks 2-12 and the baseline assessment week.
